# Social Pharmacy Research in Copenhagen—Maintaining a Broad Approach

**DOI:** 10.3390/pharmacy4010011

**Published:** 2016-02-02

**Authors:** Sofia Kälvemark Sporrong, Lotte Stig Nørgaard, Helle Wallach-Kildemoes, Lourdes Cantarero-Arévalo, Susanne Kaae

**Affiliations:** Department of Pharmacy, Faculty of Health and Medical Sciences, University of Copenhagen, Universitetsparken 2, 2100 København Ø, Denmark; lotte.norgaard@sund.ku.dk (L.N.); helle.wallach@sund.ku.dk (H.W.-K.); lou.cantarero@sund.ku.dk (L.C.-A.); susanne.kaae@sund.ku.dk (S.K.)

**Keywords:** social pharmacy, Copenhagen, research approach

## Abstract

Social Pharmacy (SP) is a multidisciplinary field to promote the adequate use of medicine. The field of SP is increasingly important due to a numbers of new trends all posing challenges to society. The SP group at the University of Copenhagen has for several years used a broad approach to SP teaching and research, often illustrated by the four levels: individual, group, organizational, and societal. In this paper the relevance of maintaining a broad approach to SP research is argued for and examples of the importance of such type of research is presented.

## 1. Introduction

Overall, social pharmacy (SP) can be said to promote the adequate use of medicine. SP is a multidisciplinary field that applies social, humanistic and natural sciences—covering areas such as sociology, anthropology, psychology, philosophy, economics, epidemiology, health, and medicine—to pharmaceuticals and related areas. For both research and teaching, SP typically borrows research methods and theories from these disciplines to cover different perspectives of medicine use in society, e.g., the patient/consumer, profession, society, and drug industry. The field of SP is increasingly important due to developments like new expensive medicines, unequal access to medicines worldwide, the strive for shared decision making, increased self-care in the developed world, the changing role of pharmacies and pharmacists in society, and the increasing use of medicines in developed countries and emerging markets. All of these factors pose new challenges to society, and SP can be part of highlighting, problematizing, putting into perspective and solving problems connected to these challenges. 

In Denmark, SP has existed as an independent research field and teaching discipline since 1989, and has since then opted for a broad approach to SP. This is for several reasons, not least of which is to educate future pharmacists and pharmaceutical scientists to be well-prepared for (the Danish) society. Students from education programs (bachelor and master of science in pharmacy, master in pharmaceutical science) in Copenhagen typically work in community pharmacies, hospitals, authorities, and, to a large degree, in the pharmaceutical industry. Therefore, highly different competencies are needed for pharmacists and pharmaceutical scientists, ranging from interactions with individual patients at the pharmacy counter to working to achieve market approval of new medicines.

Additionally in regard to research, we use a broad approach to SP. We often describe it as having four levels: individual, group, organizational, and societal. The specific distinctions between these levels are inspired by a model that illustrates the hierarchy of natural systems and their applications in social and behavioural pharmacy described by Mount [1]. The different levels and the use of various social science and humanistic perspectives in relation to those different levels help us understand and obtain a fuller picture of the role of pharmaceuticals and related issues in society.

We argue that teaching and research at each level bring value to society. Maintaining a broad approach gives strength to SP as a flexible field that is capable of adapting the research agenda to current issues, e.g., policy changes, new services and new behaviours. However, this broadness also creates challenges as a vast amount of perspectives, theories and methodologies are embraced. It is a challenge for SP to master these different disciplines in a sound and proper way without being overly superficial. 

Recently, we have experienced an increasing demand for specialization and superficial (quantitative) “productivity” within the field of SP, e.g., in relation to stakeholder interests (e.g., governmental cut-backs on education) and external funding. This demand can potentially influence which research areas and levels are prioritized and could lead to highly relevant perspectives being neglected. 

In this paper, we will show some examples of our research to argue the importance of maintaining a broad approach to SP. This paper can be used as a starting point to discuss how to best promote SP research now and in the future.

## 2. Examples and Discussion

### 2.1. Individual Level

Research at the individual level includes qualitative narrative studies. Through anthropological field work (repeated participant observations and interviews with a few participants), the use of prescription stimulants for enhancement was studied. How individuals manage different aspects of their lives—as students or in social contexts—was studied in the context of how they use and talk about stimulants (e.g., how time management could be improved or pleasure could be obtained from otherwise tedious tasks through the use of stimulants). This improved our understanding of the current role of medicines in society, specifically in regards to how medicines are consumed for new purposes that generate new ethical and societal challenges [2]. 

### 2.2. Group Level 

Much SP research is conducted at the group level. These studies are quantitative, e.g., epidemiological, and qualitative, e.g., using semi-structured interviews. The aims of these studies are typically to identify medicine-related characteristics, such as behaviours, knowledge, perceptions and attitudes, of different groups, such as patients and pharmacists. A study of regional differences in the prescription of Attention Deficit Hyperactivity Disorder (ADHD) medication for children showed that children of socially disadvantaged parents were more frequently prescribed ADHD medication in regions with high prescription rates [3]. This example shows how pharmaco-epidemiological research provides documentation of not only differences in medicine consumption by different patient groups but also prescription patterns. It also shows that socio-demographic factors still impact drug prescription, potentially generating not only inequalities but also non-rational use of medicines for certain groups.

### 2.3. Organizational Level

Studies at the organizational level often employ the same methods as those at the group level but in a different context (*i.e.*, the study of organizational features of pharmacies, hospitals, *etc.*). The aims of these studies include exploring the important factors for current professional practices and the decisions involved in those practices and studying inter-professional relations. 

The organizational context of the provision of a pharmaceutical service by pharmacies was studied using the method triangulation of observations and different types of interviews. The studies revealed that the implementation of a cognitive service is a highly complex phenomenon involving: alignment of interests between pharmacy owners and employees, the leadership style of the pharmacy owner, emphasis on service when conducting daily tasks, balance between collective/individual training of pharmacy staff, and emphatic communication skills in relation to patients, among other factors [4,5,6,7]. These factors can be directly translated into activities to improve service provision.

### 2.4. Societal Level

Finally, research at the societal level uses many of the same methods used at the organizational level; however, also document analysis is an important method used in research at the societal level. For example, an investigation into the history of the interplay between Danish politicians and pharmacy owners when forming pharmacy policy was conducted by tracing the history of the development and implementation of pharmacy law. These studies used different types of documentary material showing how politicians (kings) in the 17th century eventually had to accept that pharmacist participation was necessary for making a set of regulations that would work in practice [8]. This recognition formed the basis of the Danish tradition of consulting with pharmacy owners before putting pharmacy law into practice. This historic investigation has been shown to be capable of explaining the procedure of how pharmacy regulations are formed also in modern times; thus, it has an important explanatory capacity [9].

After presenting these examples, it should also be noted that the research projects sometimes cover many or all of these levels. One such study was a study of the Danish automated dose dispensing scheme (ADDS), which was carried out in cooperation with researchers from the Danish Southern University and the Danish College of Pharmacy Practice [10,11]. The study consisted of a literature review, and studies of persons impacted by the ADDS (including patients). It investigated participants’ experiences with the implementation of the ADDS, their opinions and perceptions of its activities, and their perceptions of the consequences of and prerequisites for the implementation of the ADDS [12,13]. Additionally, a survey was conducted of the use of the ADDS and its future potential. The strength of carrying out a study on all four levels was that the results from the different levels provided substantial and variable input on the research to be carried out on the other levels. Information that would have been insufficiently addressed had the data been collected from only a single level. For instance, the analysis of the socio-demographic characteristics of the typical Danish ADDS-user [12] strongly influenced the selection of interviewees for the patient study [13]. The study on all four levels provided broader perspectives to obtain a fuller picture of the ADDS.

## 3. Conclusions

The above-presented examples of research from our department may seem un-related to each other and could be argued to be fragmentary. However, we believe that working on different levels and over many disciplines is necessary to obtain a fuller and more nuanced picture of the role of medicines in society. Thus, maintaining a broad approach to SP research and researched-based teaching is important due to the variety and fruitfulness of the results of these broad approaches and the abilities of these broad approaches to adapt to changes in society. Furthermore, studies carried out on the same topic at different levels can generate new insights. The broad perspective we argue for ensures that SP research is relevant for patients, professionals, organizations and society. We find that maintaining a broad perspective is not a choice but an obligation. Based on our experiences and perspectives, we look forward to discussing the benefits and challenges of different approaches of conducting SP research with other researchers to develop the field further for the benefit of all.
